# Exploring the mechanism by which tourists’ perceived value influences revisit intention in sustainable gardens: A case study of KICG-sustainable garden, Shanghai

**DOI:** 10.1371/journal.pone.0338508

**Published:** 2026-01-07

**Authors:** Jiajun Chen, Guanxi Chen

**Affiliations:** 1 School of Business, Nantong Institute of Technology, Nantong, China; 2 School of Design, NingboTech University, Ningbo, China; Guilin University of Technology, CHINA

## Abstract

Sustainable gardens have emerged as innovative models of urban tourism that integrate ecological aesthetics, participatory design, and community well-being. This study explores how tourists’ perceived value influences revisit intention and introduces two experiential moderators—narrative transportation and empowerment capability—to uncover the underlying psychological and participatory mechanisms that drive behavioral intention. A mixed-methods approach was employed, combining grounded theory interviews to develop the conceptual framework with a large-scale survey analyzed using structural equation modeling (SmartPLS). The results demonstrate that perceived value positively affects revisit intention through tourist satisfaction, while narrative transportation and empowerment capability significantly strengthen these relationships by enhancing emotional immersion and participatory engagement. These findings advance sustainable tourism theory by highlighting the dual-path moderating effects of storytelling and empowerment in shaping visitor behavior. Practically, the study provides actionable insights for sustainable garden management, emphasizing the importance of immersive narratives and participatory empowerment in fostering long-term tourist loyalty and sustainable destination development.

## 1 Introduction

In the post-pandemic era of urban regeneration, sustainable gardens—integrating ecological aesthetics, environmental education, and participatory design—have emerged as new engines of sustainable urban tourism [1,2]. These multifunctional environments not only enhance biodiversity and urban livability but also offer visitors immersive experiences that link ecological awareness with community values and narrative expression [3,4]. As global cities increasingly adopt low-carbon and inclusive cultural models, sustainable gardens have become laboratories for testing how environmental storytelling and participatory practices can foster public engagement and sustainable behavior.

However, despite their growing visibility in urban regeneration agendas, existing tourism research has not kept pace with this emerging phenomenon. Previous studies on sustainable tourism have predominantly emphasized infrastructure, spatial design, and environmental performance, while neglecting the behavioral and psychological dimensions that determine whether visitors develop long-term engagement with such spaces [5,6]. This disconnect has resulted in limited understanding of how tourists perceive, internalize, and act upon sustainability-oriented experiences in these hybrid public–tourism environments.

Despite incremental progress, the behavioral mechanisms shaping tourists’ experiences and revisit intentions in sustainable gardens remain insufficiently explored. Existing research on community-based tourism (CBT) has largely addressed three focal areas—community empowerment and sustainable development [7–9], tourist co-creation and value experience [10,11], and the influence of satisfaction and emotional identification on loyalty [12]—yet these studies are predominantly rooted in rural or commercial contexts. In contrast, sustainable gardens represent urban, mission-driven spaces that combine ecological consciousness, participatory governance, and cultural education, where the motivational structure of visitors differs substantially from traditional leisure environments. This shift underscores an urgent need to establish a new behavioral framework that captures the cognitive, emotional, and participatory dynamics of sustainable tourism in metropolitan settings.

Among these factors, perceived value has long been recognized as a central determinant of tourist satisfaction and loyalty [13,14]. Yet, its behavioral implications in non-commercial, educational tourism spaces remain unclear. While many scholars have confirmed the positive relationship between perceived value and revisit intention, few have investigated how this relationship operates under conditions emphasizing environmental learning, collective participation, and symbolic meaning—core features of sustainable gardens [15]. In particular, how perceived value translates into behavioral intention through emotional and participatory experiences remains under-theorized, leaving a critical gap in understanding visitors’ motivations toward sustainable destinations.

To clarify these processes, this study introduces two experiential constructs—narrative transportation and empowerment capability—as the psychological bridges linking perception and behavior. Narrative transportation captures the degree of emotional immersion and identification in environmental storytelling, while empowerment capability reflects tourists’ sense of autonomy, participation, and influence during co-creative experiences. By integrating these two constructs into the perceived value–behavior framework, this study responds to contemporary demands for understanding post-pandemic tourism behaviors that are characterized by deeper psychological involvement, self-expression, and shared responsibility for sustainability.

Accordingly, this study aims to examine how tourists’ perceived value influences their revisit intention in sustainable garden contexts, and to clarify the moderating roles of narrative transportation and empowerment capability. The study addresses two key research questions:

(1) How does tourists’ perceived value affect revisit intention in sustainable garden tourism?
(2) How do narrative transportation and empowerment capability interact with this relationship?

To answer these questions, a mixed-methods approach was employed, combining qualitative grounded theory to inductively develop a conceptual model and quantitative structural equation modeling (SEM) to test it empirically. This integrative design advances the behavioral study of sustainable tourism by connecting cognitive evaluation (perceived value), emotional immersion (narrative transportation), and participatory agency (empowerment capability) into a coherent theoretical framework. Ultimately, this research provides a timely response to contemporary conditions of urban sustainability transformation, offering both theoretical innovation and practical guidance for sustainable destination design and management.

## 2 Literature review

### 2.1 Tourists’ perceived value and revisit intention

Perceived value is commonly defined as the overall evaluation formed by tourists after a travel experience, based on their subjective comparison between the benefits gained and the costs invested (e.g., time, money, effort). It is widely regarded as a key predictor of satisfaction and behavioral intentions [16]. In the context of sustainable and community-based tourism, perceived value extends beyond functional and economic dimensions to include emotional, symbolic, and cognitive aspects [6]. Research suggests that in ecotourism settings, emotional fulfillment and cognitive stimulation enhance tourists’ sense of belonging and loyalty [17].

Particularly in sustainable gardens—where multisensory engagement and participatory interaction are emphasized—perceived value often encompasses aesthetic appreciation, personal reflection, and community interaction. However, little empirical research has systematically examined how such perceived value affects tourists’ revisitation behaviors [18]. Existing studies tend to focus on natural or heritage tourism, with relatively limited attention to emerging forms of garden tourism that integrate symbolic meaning with urban leisure [19,20]. In this study, perceived value is defined as the overall evaluation formed by tourists based on their subjective comparison between the benefits gained and the costs invested, and is positioned as the core cognitive driver: we will examine its relationship with satisfaction and revisit intention, to clarify its direct and indirect effects in the context of urban sustainable gardens.

### 2.2 Narrative transportation and tourist behavior

Narrative transportation refers to a psychological state of immersive engagement that occurs when individuals are exposed to a narrative, characterized by attentional focus, identification with characters, and emotional resonance [21,22]. In tourism studies, this concept has been used to explain how destination storytelling—through guided interpretation, spatial design, or digital media—affects tourists’ memory, satisfaction, and loyalty [23]. Story-rich promotional materials, such as short-form videos, have been shown to enhance favorable attitudes toward destinations and increase visit intentions [24–26].

Nevertheless, the theoretical integration of narrative transportation as a moderating mechanism between perceived value and behavioral outcomes remains underdeveloped. Most existing research emphasizes its application in marketing communication and brand building [27], with limited focus on how tourists engage with narrative-driven experiences in participatory environments like sustainable gardens. Thus, narrative transportation offers a valuable but underutilized theoretical lens to explain the connection between tourist perceptions and behavioral intentions.In this study, narrative transportation is viewed as an in-situ emotional-meaning-making mechanism: we focus on how it influences the strength of the relationship between “perceived value” and subsequent evaluations/intention, defining the actual role of narrative in sustainable garden experiences.

### 2.3 Empowerment capability and revisit motivation

Empowerment capability in tourism refers to the degree to which tourists perceive autonomy, expressive agency, and social influence during participatory experiences [28]. Although originally applied to assess community empowerment in CBT contexts [29], the concept has recently been extended to understand tourists’ subjective experiences in co-creative and service interaction settings [30]. Studies have shown that when tourists perceive higher empowerment, their psychological belonging, motivation, and satisfaction improve significantly, thereby enhancing revisit intention [31,32].

However, in experience-centered sustainable garden tourism—such as community workshops, collaborative exhibitions, and environmental education—how tourists’ sense of empowerment influences their subsequent behaviors remains underexplored. Through active participation in gardening, offering feedback, or contributing to event planning, tourists may develop a sense of respect and influence. This process may serve as a key factor linking satisfaction to revisit intention.In this study, empowerment capability is defined as tourists’ perceived autonomy, expression, and influence, and we will investigate how it strengthens the relationship between satisfaction and revisit intention, with particular attention to how participatory roles and co-creation opportunities may enhance positive behavioral tendencies.

## 3 Case selection

The selection of the Knowledge and Innovation Community Garden (KICG-Sustainable Garden) in Shanghai as the case study site for this research is based on its representativeness, relevance to the research theme, and its strong alignment with the core principles of community-based sustainable tourism.

First, Shanghai is one of the most prominent cities in China in terms of urban regeneration and sustainable practice. Since the release of the Implementation Measures for Urban Regeneration in Shanghai in 2015, the municipal government has actively encouraged multi-stakeholder participation in ecological renovation and urban renewal [33]. Against this policy backdrop, the KICG project emerged, emphasizing ecological restoration, civic engagement, and sustainable living. In 2018, it was recognized as one of the “Top Ten Social Innovation Practices in Shanghai, China” [34], demonstrating its significant public influence.

**Fig 1:**
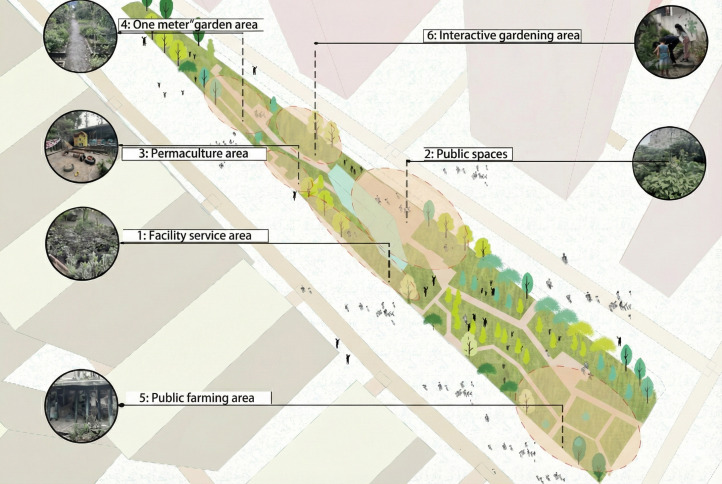
KICG-sustainable garden (created by the authors from field observation).

Second, as a model for green urban transformation, KICG-Sustainable Garden integrates ecological aesthetics, community participation, and urban leisure outreach. The garden spans 2,200 square meters and is divided into six functional zones: (1) Facility service area, (2) Public spaces, (3) Permaculture area, (4) “One Meter” garden area, (5) Public farming area, and (6) Interactive gardening area (Fig 1). Every weekend and public holiday, the site hosts a wide array of participatory programs, including guided tours, hands-on gardening workshops, and environmental education sessions—providing rich empirical material for investigating tourist experiences and behavioral patterns.

Third, KICG-Sustainable Garden has gradually evolved from a community-focused ecological education space into a micro-tourism destination that attracts a broader visitor base. By leveraging a proprietary WeChat mini-program to release activity schedules and enable online reservations, the garden continuously draws interest from individuals and families seeking nature-based experiences, environmental learning, artisanal crafting, and parent-child interaction. This transition has turned the garden into a quintessential setting for “urban pastoral” micro-tourism, appealing not only to local residents but also to non-local tourists. As a result, KICG has become a popular site for user-generated recommendations and cultural check-ins, highlighting its dual appeal for both community development and sustainable tourism. Its high degree of integration between community engagement, participatory experience, and tourism functionality offers a representative and practice-based case for exploring the mechanisms of perceived value and revisit intention in sustainable tourism contexts.

## 4 Materials and methods

### 4.1 Research procedure and samples

This study adopted a mixed-methods research design integrating qualitative exploration and quantitative validation to examine how tourists’ perceived value influences revisit intention in the context of sustainable garden tourism. The qualitative phase aimed to explore the types and formation processes of tourists’ perceived value, focusing on two core questions: how tourists perceive and experience value during their visit, and how these perceived values shape their intention to revisit. To address these questions, a semi-structured interview guide was designed with input from two tourism management and sustainable development experts, two doctoral students specializing in tourist behavior, one garden operations planner, and three experienced visitors. The interviews were organized around four thematic areas: the process of tourist interaction with the sustainable garden, the types and sources of perceived value, the influence of value perception on behavioral tendencies, and tourists’ receptiveness to the garden’s sustainability philosophy. Fieldwork was conducted in situ at the KICG Sustainable Garden between July 22 and July 29, 2024, to capture real-time experiences. A total of 36 participants, including both first-time and repeat visitors, were recruited through purposive sampling and participated in either group discussions or individual interviews. All procedures adhered to ethical standards, with participants informed about voluntary participation and data confidentiality, and written consent obtained. The interview process followed a structured three-stage procedure—preparation, implementation, and post-processing. The collected audio data amounted to 548 minutes and 142,000 words of transcripts. Grounded theory was used for coding and analysis, supported by NVivo 14 software to facilitate open, axial, and selective coding. The data reached theoretical saturation when no new themes emerged from the final four interviews. The qualitative results guided the conceptual model and provided empirical grounding for the subsequent quantitative phase.

The quantitative phase employed a structured questionnaire survey to test the proposed model. Data were collected on-site at the same location from September 28 to October 28, 2024, yielding 543 valid questionnaires. The survey targeted adult visitors (aged 18 and above) who had completed at least one full garden experience and consented to participate voluntarily. The sample was balanced across gender and education level, with the majority aged between 20 and 40 years, representing the main demographic of sustainable garden visitors. The survey ensured anonymity and confidentiality throughout the process. The study passed university ethics review; all data are used solely for academic research and are stored strictly separate from any personally identifying information.

The questionnaire was originally developed in English and then translated into Chinese to ensure linguistic and cultural appropriateness for local respondents. The translation process followed the standard back-translation procedure [35]. Two bilingual experts independently translated the English version into Chinese, and a third bilingual researcher—unfamiliar with the original questionnaire—back-translated it into English. Any discrepancies were discussed and reconciled by the research team to ensure semantic equivalence and conceptual consistency. A pilot test with 30 Chinese respondents was conducted to confirm clarity, readability, and contextual accuracy, with minor wording adjustments made based on feedback prior to final survey distribution.

To enhance the generalizability and representativeness of the findings, the sampling strategy was designed to capture a diverse range of visitors who represent the core demographic of sustainable garden tourism in urban China. The KICG Sustainable Garden was selected as a typical case because it attracts a heterogeneous mix of visitors, including families, young professionals, students, and retirees interested in environmental education and community-based recreation. During both the qualitative and quantitative phases, efforts were made to include participants of different gender, age, education, and visitation frequency. Recent tourism-methodology studies note that sample selection must align with the target population to enhance external validity [36] and that purposeful sampling can still be credible when paired with transparency about representativeness and methodological rigor [37]. Although the sampling approach in this study was non-probabilistic, it followed the principle of purposeful heterogeneity widely applied in contemporary tourism behavior research [38]. This approach seeks to capture diverse participant profiles to enhance contextual representativeness within a specific experiential setting. Therefore, while the findings are not intended for statistical generalization across all tourist populations, they are analytically generalizable to comparable urban sustainable tourism contexts that exhibit similar ecological, leisure, and participatory characteristics [37].

### 4.2 Research instruments

The questionnaire consisted of two major sections: demographic information (gender, age, education level, and monthly income) and measurement scales for the five latent constructs, all assessed using a 7-point Likert scale (1 = strongly disagree, 7 = strongly agree).

Perceived value was modeled as a three-dimension construct—emotional value (3 items), service value (3 items), cognitive value (3 items)—adapted from Jeong & Kim (2020) [14] and Özkan et al. (2020) [39]. Empowerment capability comprised political empowerment (3 items), psychological empowerment (3 items), and social empowerment (3 items), adapted from Joo et al. (2020) [28], Scheyvens & van (2021) [40], and Noordink et al. (2021) [41]. Narrative transportation was captured via narrative content (3 items) and narrative effect (3 items) based on Cao et al. (2021) [24]and Yang & Kang (2021) [42]. Tourist satisfaction (3 items) and revisit intention (3 items) were adapted from Jeong & Kim (2020) [14], Isa et.al., (2020) [43].

All items underwent expert review to ensure content validity using the Item–Objective Congruence (IOC) method [44],with IOC scores exceeding 0.70. A pilot test involving 30 participants was conducted to examine reliability and clarity. The results demonstrated strong internal consistency, with Cronbach’s α values ranging from 0.82 to 0.91, surpassing the recommended 0.70 threshold [45]. Minor wording revisions were made based on pilot feedback to improve comprehension and contextual accuracy. The final instrument was thus validated for both reliability and construct validity, providing a robust foundation for subsequent hypothesis testing using structural equation modeling (SEM) in SmartPLS 4. Detailed item descriptions are presented in Table 1.

**Table 1:** Summary of the construct items.

Initial dimension	Code and Item description
Perceived Value	
Emotional (EMO)	**EMO1:** Through the shared practice of the sustainable garden, you have grown to love community life more.
	**EMO2:** You feel proud of participating in the sustainable garden project.
	**EMO3**: You find participating in the sustainable garden to be enjoyable and fun.
Service (SER)	**SER1:** The project organizers have a high level of professionalism.
	**SER2:** The project organizers are experienced.
	**SER3:** The project organizers are serious and attentive about the project planning and implementation process.
Cognitive (COG)	**COG1:** The sustainable garden has popularized the concept of sustainability, allowing more people to understand and appreciate its importance.
	**COG2:** Everyone has an obligation to change their mindset and work together to achieve the sustainable development of the garden.
	**COG3:** After experiencing the project, you have gained a deeper insight into the essence of the sustainable garden.
Empowerment	
Political (POL)	**POL1:** Your opinions influence the development of the project.
	**POL2:** You participated in the formulation of the project plan.
	**POL3:** You were involved in the development planning of the sustainable garden.
psychological (PSY)	**PSY1:** You have learned new things and broadened your knowledge through the sustainable garden.
	**PSY2:** You have had new and different experiences through participating in the sustainable garden project.
	**PSY3:** Your participation in the project leads you to reflect on yourself and aspire to become a better person.
Social (SOC)	**SOC1:** The sustainable garden has strengthened interpersonal relationships within the team.
	**SOC2:** The sustainable garden project has provided you with opportunities and venues to participate in public activities.
	**SOC3:** The sustainable garden project has motivated you to participate in public activities.
Narrative Transportation	
Content (CON)	**CON1:** While experiencing the project, you can easily sense the events happening in the setting.
	**CON2:** During the project experience, my thoughts are captivated by the conversations within the activities.
	**CON3:** I vividly remember my experiences participating in the sustainable garden project.
Effects (EFF)	**EFF1:** You will continue to visit the sustainable garden to experience it further.
	**EFF2:** You will participate in other social activities related to sustainable garden concepts that you encounter.
	**EFF3:** After participating in the project, you will carry forward the sustainable garden concept in your community or future plans.
Revisit Intention (RI)	**RI1:** If given the opportunity, you would participate in project activities here again.
	**RI2:** You will actively recommend your friends, family, and colleagues to visit the sustainable garden.
	**RI3:** Overall, you are eager to come back and experience it again.
Participant Satisfaction (PS)	**TS1:** You are satisfied with your overall participation experience in the sustainable garden.
	**TS2:** The facilities, services, and project experiences at the sustainable garden met your expectations.
	**TS3:** You are satisfied with the professional attitude of the sustainable garden project organizers.

### 4.3 Qualitative study on tourists’ perceived value in sustainable gardens

#### 4.3.1 Research procedure and data collection.

The qualitative phase of this study was designed to explore how tourists perceive and experience value in sustainable gardens and how such perceptions shape their revisit intention. Semi-structured interviews were conducted in situ at the KICG Sustainable Garden to capture authentic, real-time experiences. The interview guide, developed collaboratively with academic experts and practitioners, covered four key themes: tourist interaction processes, sources of perceived value, behavioral influences, and responses to sustainability messages.

A total of 36 participants were recruited through purposive sampling and engaged in either group discussions or individual interviews(Table 2). All procedures adhered to research ethics standards, with participants informed about voluntary participation and confidentiality, and written consent obtained. Interviews were audio-recorded, transcribed, and analyzed using NVivo 14 following grounded theory coding procedures (open, axial, and selective coding). Data saturation was achieved when no new categories emerged in the final interviews, yielding a total of 548 minutes of recordings and 142,000 words of transcripts.

**Table 2:** List of intervew subject information.

No	Gender	Age	Occupation	No.	Gender	Age	Occupation
1	M	49	Employee	22	M	18	Student
2	F	42	Employee	23	F	36	Self-employed
3	F	65	Retired	24	F	32	Self-employed
4	M	36	Government Employee	25	M	49	Employee
5	M	45	Employee	26	M	20	Student
6	F	39	Employee	27	M	41	Self-employed
7	F	22	Student	28	F	50	Government Employee
8	F	39	Government Employee	29	F	55	Self-employed
9	F	41	Employee	30	M	39	Employee
10	M	39	Self-employed	31	M	18	Student
11	F	38	Government	32	F	20	Student
12	M	49	Self-employed	33	M	21	Student
13	F	42	Employee	34	F	33	Self-employed
14	M	46	Self-employed	35	F	55	Government Employee
15	F	49	Government Employee	36	M	60	Retired
16	M	42	Employee				
17	F	65	Retired				
18	M	57	Employee				
19	M	45	Government Employee				
20	F	41	Self-employed				
21	F	27	Employee				

#### 4.3.2 Grounded theory analysis and theoretical exploration.

This study adopted procedural grounded theory as the methodological tool for its qualitative analysis. The choice of this approach was based on two primary considerations. First, grounded theory is particularly well-suited for exploring individuals’ cognitive and behavioral processes within specific contexts. It emphasizes the generation of theory from empirical data, aligning closely with the objective of this research, which is to understand tourists’ perceptions of value in sustainable gardens and to explore the mechanisms through which such perceptions influence behavior. Second, the concept of perceived value in the context of sustainable garden tourism remains a developing construct. Its internal structure and functional pathways require further clarification and theoretical refinement.

Therefore, grounded theory was employed to conduct an abstract analysis of tourists’ experiential and perceptual processes. The analysis followed the classic three-stage procedure of open coding, axial coding, and selective coding. Through this step-by-step process, the study aimed to identify key categories and progressively extract core theoretical constructs.

(1) Open Coding. The open coding phase of this study involved line-by-line analysis of the original interview transcripts to identify preliminary concepts directly grounded in the data. Through iterative comparison and conceptual abstraction, the research team constructed a theoretical foundation closely aligned with the research questions. During the coding process, a stepwise inductive approach was employed to ensure a strong connection between emerging concepts and the raw data, thereby maintaining the integrity and systematic nature of the information.

As a result, a total of 312 initial codes were extracted from the interview data, which were further refined and categorized into 29 preliminary concepts. These concepts served as the basis for the subsequent axial coding and the development of the theoretical model. Illustrative examples of the coding process, from raw data to conceptual extraction, are presented in Table 3.

**Table 3:** Example of the formation process from raw materials to concepts (partial).

Original Data Excerpt	Initial Concept	Category
**a1:** This garden gives off a strong sense of being green and eco-friendly. It’s not just a place to look at flowers. **a2:** The layout is well planned. Facilities like restrooms and seating areas are quite convenient. **a3:** I’m usually very busy, but being here made me feel instantly relaxed. **a4:** The activities here are highly immersive. Tourists can really participate in the experience, unlike typical sightseeing. It feels more alive, more connected to nature and daily life. **a5:** I initially thought it would just be a place to walk around, but I was surprised by how many interactive and educational elements it had.	**a1:** Tourists perceive the garden as having ecological value and serving as a medium for conveying green and sustainable concepts. **a2:** Tourists experience functional satisfaction from the spatial layout, facilities, and services provided. **a3:** Tourists report emotional pleasure, relaxation, or inspiration during the experience. **a4:** Tourists appreciate the overall cultural atmosphere and aesthetic presentation of the garden. **a5:** Tourists perceive a sense of value that exceeds their initial expectations.	A1:Perceived Value
**a6:** The staff were very friendly, and the environment was well maintained. You can tell they put effort into it. **a7:** Some of the interactive activities really exceeded my expectations. The kids enjoyed them a lot too. **a8:** After participating in the hands-on activities, I felt even more positively about the place.	**a6:** Tourists express positive evaluations regarding staff service and the cleanliness of the environment. **a7:** The experience content exceeds expectations, and the garden’s programs are considered engaging and enjoyable. **a8:** Positive feelings generated through interactive experiences enhance tourists’ overall satisfaction.	A2:Participant Satisfaction
**a9:** The information boards were clear, and I could understand what the garden was trying to convey. **a10:** Each area had a theme with simple explanations, which made it easy to follow. **a11:** When the guide mentioned that this used to be an abandoned site, it suddenly felt very meaningful. **a12:** After my visit, I even told my friends that this place has a strong environmental message and is really interesting.	**a9:** Tourists gain an understanding of the garden’s core concepts through signage or interpretive explanations. **a10:** Tourists acknowledge that the garden offers highly experiential programs and clear information delivery. **a11:** Narrative elements enhance tourists’ emotional engagement and sense of identification with the garden. **a12:** After the visit, tourists actively communicate the garden’s concepts to others, demonstrating a strong sense of narrative identification.	A3:Narrative Transportation
**a13:** I joined an eco-workshop and made something myself. It was much more fun than just walking around. **a14:** They had a feedback wall. I left some suggestions and felt that my opinion was respected. **a15:** You can stay as long as you want in any area. It’s not one of those places where you’re forced to follow a fixed path. **a16:** They asked me to interpret the craft I made. It made me feel like a real artisan. **a17:** I once suggested they add a sunshade, and on my next visit, I saw they had actually done it. It felt like my input really mattered.	**a13:** Tourists feel a sense of participation through engagement in interactive programs or experiential activities. **a14:** Tourists are given opportunities to express opinions or suggestions during the experience. **a15:** The garden’s flexible design allows for a high degree of autonomy in choosing spaces and activities. **a16:** Tourists experience a sense of agency during interactions, shifting from passive observers to active participants. **a17:** Tourists believe their participation has had, or could have, a tangible or potential impact on the garden’s content or operations.	A4:Empowerment Capability

(2) Axial Coding. The purpose of axial coding is to identify relationships among the initial categories and to further clarify their underlying meanings. In this phase, the study drew upon the results of open coding and applied inductive analytical methods to systematically organize the categories and examine their interconnections. The goal was to establish theoretical linkages among the core categories. Through repeated comparison and iterative refinement, a total of 29 initial concepts were grouped and consolidated into 8 subcategories. This process enhanced the clarity of the data structure and contributed to the development of a coherent theoretical framework (Table 4).

**Table 4:** Comparison table between subcategories and initial concepts.

Category	Initial Concept
**A1:**Perceived Value	**a1:** Tourists perceive the garden as having ecological value and as a medium for promoting green concepts. **a2:** Tourists experience functional satisfaction with the spatial layout, facilities, and services. **a3:** Tourists gain emotional pleasure, relaxation, or inspiration during the experience. **a4:** Tourists appreciate the garden’s cultural atmosphere and aesthetic presentation. **a5:** Tourists perceive a sense of value that exceeds their initial expectations.
**A2:**Participant Satisfaction	**a6:** Tourists express positive evaluations of staff service and environmental cleanliness. **a7:** The experience content exceeds expectations, and the garden’s programs are found to be engaging. **a8:** Positive feelings during interaction enhance tourists’ overall satisfaction.
**A3:**Narrative Transportation	**a9:** Tourists gain an understanding of the garden’s concepts through signage or guided interpretation. **a10:** Tourists recognize that the garden’s programs are highly experiential and convey information clearly. **a11:** Narrative elements enhance tourists’ emotional engagement and sense of identification. **a12:** After the visit, tourists actively communicate the garden’s values to others, indicating strong narrative identification.
**A4:**Empowerment Capability	**a13:** Tourists feel a sense of participation through engaging in interactive programs or hands-on activities. **a14:** Tourists are provided with opportunities to express opinions or suggestions during the experience. **a15:** The garden offers flexible spaces and high autonomy for tourists to make their own choices. **a16:** Tourists experience a sense of agency during interactions, shifting from passive observers to active participants. **a17:** Tourists believe their participation has had, or could have, an actual or potential impact on the garden’s content or operations.
**A5:**Revisit Intention	**a18:** Tourists express a clear intention to revisit the garden. **a19:** Tourists are willing to recommend the garden to friends or revisit with companions. **a20:** Tourists express interest in seasonal changes or content updates in the garden.
**A6**:Environmental Atmosphere Identification	**a21:** Tourists show strong identification with the natural environment and spatial atmosphere. **a22:** The garden space is perceived as safe, comfortable, and of high quality. **a23:** Tourists regard the garden as a rare ecological space within the city. **a24:** Tourists resonate with the ecological aesthetics promoted by the garden.
**A7:**Social Relations	**a25:** Tourists frequently and positively engage in social interaction during the experience. **a26:** The garden serves as a shared space for family, parent–child, or friend-based interactions.
**A8:**Education and Learning	**a27:** Tourists acquire knowledge related to sustainability or ecology. **a28:** Tourists acknowledge the garden’s role in public environmental education. **a29:** The experience evokes tourists’ awareness or reflection on low-carbon urban living.

(3) Selective Coding. In the selective coding phase, this study focused on analyzing the theoretical relationships among the major categories and identifying the core category based on the integration of these relationships.

**Fig 2:**
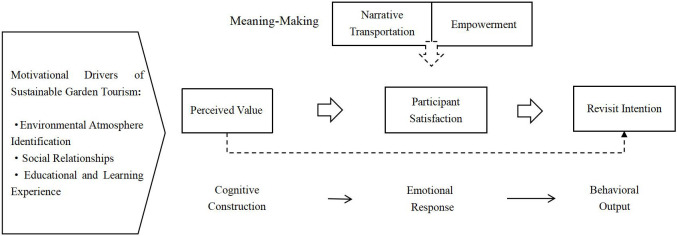
Theoretical framework of the influence of tourists’perceived value on revisit intention in sustainable gardens.

By abstracting and synthesizing the results of axial coding, five major categories were identified: cognitive construction, emotional response, meaning construction, behavioral output, and the driving mechanisms behind the emergence of sustainable garden tourism experiences. These categories were carefully compared with the original interview data to ensure consistency and theoretical saturation. Through this comparison, the relational mechanisms between the five major categories and the eight subcategories were identified (Table 5). Based on this analysis, a conceptual model illustrating the underlying mechanisms was developed (Fig 2).

**Table 5:** Category relationship and category connotation between main and subeategories.

Main Category	Subcategory	Category Concept
**B1** Cognitive Construction	A1:Perceived Value	Tourists’ overall evaluation of their experience in a sustainable garden is largely shaped by their comprehensive perception of the functional, emotional, ecological, and cultural values provided by the garden.
**B2** Emotional Response	A2:Participant Satisfaction	Tourists’ subjective level of satisfaction, formed through service encounters, activity participation, and environmental perception during the visit, reflects the degree of alignment between their expectations and actual experiences.
**B3** Meaning Construction	A3: Narrative Transportation	The sustainable garden conveys its core concepts and cultural narratives through spatial design, interpretive content, and symbolic systems, which in turn shape tourists’ understanding and identification with its values.
	A4:Empowerment Capability	The sense of active participation and opportunities for self-expression that tourists gain during the experience—such as co-design, interactive feedback, and content co-creation—demonstrates their agency and the extent to which they feel respected.
**B4** Behavioral Output	**A5:**Revisit Intention	Tourists’ intention to revisit the sustainable garden after completing the experience reflects the site’s sustained attractiveness, which is typically influenced by a combination of factors.
**B5** Motivational Drivers of Sustainable Garden Tourism	**A6**:Environmental Atmosphere Identification	Tourists’ psychological identification with the garden’s natural environment, spatial atmosphere, and sustainability philosophy serves as a key foundation for emotional connection and attitude formation.
	**A7:**Social Relations	The nature of tourists’ interpersonal interactions during the experience—such as with family, friends, or companions—and their influence on the perceived quality of the experience are critical for fostering collective participation and word-of-mouth dissemination.
	**A8:**Education and Learning	Tourists’ acquisition of knowledge and cognitive growth through interpretive content, interactive displays, and hands-on activities during the visit represents an important pathway for enhancing the perceived meaning and social value of the tourism experience.

### 4.4 Discussion of qualitative findings

As illustrated in Fig 2, tourists’ revisit intentions in the context of sustainable gardens are shaped by a combination of factors, including social relations, identification with the environmental atmosphere, and leisure or learning-related experiences.

From a theoretical perspective, cognitive construction, emotional response, and meaning construction are found to be interrelated with revisit intention. This study finds that tourists’ cognitive construction during their experience in the garden contributes to the generation of positive emotional responses, which in turn serve as a key foundation for the formation of revisit intention. Furthermore, meaning construction is identified as a critical condition influencing both emotional responses and the willingness to revisit. In this process, tourists’ meaning construction is particularly important, as it influences both the generation of emotional responses and the formation of revisit intention.

Perceived empowerment is reflected primarily in tourists’ sense of agency and level of participation during the experience. The study indicates that when tourists perceive themselves as having influence—through feedback mechanisms, interactive programs, or opportunities for self-expression—and feel respected within the garden setting, their intention to revisit significantly increases. Similarly, narrative transportation, defined as tourists’ psychological immersion in the experiential setting and their sense of mental “entry” into the story world, also plays a role in shaping emotional responses and behavioral intentions. In this study, empowerment is primarily reflected through tourists’ sense of autonomy and participation within the garden. When tourists feel empowered and respected through feedback mechanisms, interactive programs, or opportunities for self-expression, their revisit intention increases significantly.

Through the inductive approach of grounded theory, the qualitative research developed a theoretical framework explaining how perceived value influences revisit intention in the context of sustainable gardens. This framework reveals how perceived value, through cognitive, emotional, and participatory processes, ultimately affects tourists’ revisit intention in the context of sustainable gardens. Following the identification of these theoretical relationships, the study will proceed to a quantitative phase to further clarify the causal pathways between key variables, particularly the linkage between cognitive construction and behavioral output.

Given that grounded theory does not directly reveal causal relationships among variables, the next stage of the research will involve formulating hypotheses based on the core concepts derived from the qualitative findings—namely, perceived value, participant satisfaction, narrative transportation, perceived empowerment, and revisit intention. In the next stage, hypotheses will be formulated based on the core concepts identified in the qualitative phase and will be tested through empirical data collection and quantitative analysis to validate the causal relationships between perceived value, satisfaction, narrative transportation, empowerment, and revisit intention.

### 4.5 Model and hypotheses development

#### 4.5.1 The influence of perceived value on revisit intention in sustainable garden tourism.

Within the context of sustainable tourism, perceived value is defined as tourists’ comprehensive psychological evaluation formed after comparing the emotional, functional, environmental, and cultural benefits obtained from the tourism experience against the costs invested, such as time, money, and effort [18]. Sustainable gardens integrate ecological aesthetics, community interaction, and experiential learning, offering tourists a platform for acquiring multidimensional value [46].

Existing studies suggest that perceived value significantly influences tourist satisfaction, which in turn is a strong predictor of revisit intention [13,18]. However, few models specifically investigate how perceived value functions in immersive and community-oriented tourism experiences like sustainable gardens. Unlike traditional sightseeing, such gardens emphasize participatory interaction and a slower-paced, reflective experience.

Findings from the qualitative phase indicate that tourist satisfaction in sustainable gardens is not merely derived from basic service quality or visual impressions, but is more closely linked to emotional resonance and the construction of personal meaning during the experience. This offers theoretical insights for further exploring behavioral pathways in tourism.

Accordingly, the following hypotheses are proposed for subsequent quantitative validation

H1: Perceived value positively affects tourists’ revisit intention in the context of sustainable gardens.

H2: Tourist satisfaction mediates the relationship between perceived value and revisit intention.

#### 4.5.2 The moderating role of narrative transportation.

Narrative transportation refers to a psychological state of immersion in a story, characterized by focused attention on the plot, identification with characters, and emotional resonance [22]. In tourism research, it is considered a crucial psychological mechanism for enhancing immersion and destination attachment [25].

Recent literature highlights the importance of storytelling and narrative framing in shaping tourists’ emotional and behavioral responses toward destinations [24,47]. Narrative-rich tourism environments, such as sustainable gardens, employ symbolic cues, cultural scripts, and interpretive storytelling to help visitors construct meaningful place perceptions and memorable experiences.

Preliminary qualitative results suggest that when tourists experience strong emotional engagement and narrative comprehension during their visit, they are more likely to develop identification and satisfaction, thereby increasing their intention to revisit.

Thus, the following exploratory hypotheses are proposed

H3: Narrative transportation moderates the relationship between perceived value and revisit intention.

H4: Narrative transportation moderates the relationship between perceived value and tourist satisfaction.

#### 4.5.3 The moderating role of empowerment capability.

Empowerment capability refers to an individual’s perceived sense of autonomy, expression rights, and influence within participatory settings [28]. In the context of sustainable garden tourism, psychological empowerment involves tourists’ confidence and self-worth, social empowerment relates to their ability to form community connections, and political empowerment refers to their capacity to voice opinions and engage in decision-making [30].

The qualitative findings of this study indicate that sustainable gardens offer participatory experiences that enable tourists to engage in ecological stewardship, co-create interpretive content, and provide feedback on site design and operations. It is thus hypothesized that perceived empowerment may influence the linkage between satisfaction and subsequent behavioral intentions.

Accordingly, the following hypothesis is proposed

H5: Empowerment capability moderates the relationship between tourist satisfaction and revisit intention.

**Fig 3:**
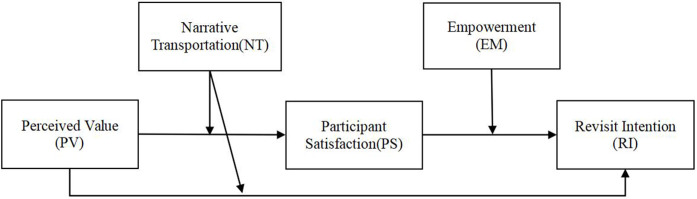
Model.

In summary, based on the results of qualitative analysis and existing theoretical insights, this study proposes a comprehensive conceptual model(Fig 3). The model includes five key constructs: perceived value, narrative transportation, participant satisfaction, revisit intention, and empowerment capability.

## 5 Testing the mechanism of tourists’ perceived value on revisit intention in sustainable gardens

This study employed Partial Least Squares Structural Equation Modeling (PLS-SEM) using SmartPLS 3.0 to test the proposed hypotheses. PLS-SEM is widely used in hospitality and service research due to its suitability for complex, formative, and higher-order models, and because it does not require strict assumptions of normal data distribution. The analysis began with a confirmatory factor analysis (CFA) using the partial least squares method, followed by hypothesis testing based on the theoretical framework.

### 5.1 Respondents’ profile characteristics

This study employed a non-probability sampling method to collect data. The finalized questionnaire was primarily distributed on-site. A total of 543 valid responses were obtained. All questionnaires were completed anonymously, and participation was entirely voluntary. The demographic characteristics of the sample are summarized in Table 6.

**Table 6:** Respondents’ demographic proﬁles.

		Frequency	%	
Gender	Make	293	53.959	
	Female	250	46.041	
Occupation	Student	18	3.315	
	Government employee	81	14.917	
	Corporate employee	228	41.989	
	Self-employed	216	39.779	
Education	High school education or below	174	32.044	
	Bachelor’s Degree	290	53.407	
	Master’s Degree and Above	79	14.549	
Age	18	4	0.737	
	19-25	8	1.473	
	26-35	69	12.707	
	36-45	298	54.880	
	46-55	134	24.678	
	≥55	30	5.525	
Monthly income	≤¥2999	152	27.993	
	¥3000-5999	202	37.201	
	¥6000-8999	138	25.414	
	≥¥9000	51	9.392	
Total		543	100.0	

According to the frequency analysis, the gender distribution was as follows: 53.959% of respondents were male (n = 293), and 46.041% were female (n = 250). Regarding occupation, 41.989% (n = 228) were corporate employees, 39.779% (n = 216) were self-employed individuals, 14.917% (n = 81) were government employees, and 3.315% (n = 18) were students.

In terms of educational background, 53.407% (n = 290) of respondents held a bachelor’s degree, 32.044% (n = 174) had a high school education or below, and 14.549% (n = 79) held a master’s degree or higher. With respect to age, the largest group was aged 36–45, accounting for 54.880% (n = 298), followed by those aged 46–55 at 24.678% (n = 134), those aged 26–35 at 12.707% (n = 69), respondents aged 55 and above at 5.525% (n = 30), and respondents aged 18 at 0.737% (n = 4).

In terms of monthly income, the largest proportion of respondents (37.201%, n = 202) reported an income between 3,000 and 5,999 RMB. This was followed by those earning 2,999 RMB or less (27.993%, n = 152), those earning between 6,000 and 8,999 RMB (25.414%, n = 138), and those with a monthly income of 9,000 RMB or more (9.392%, n = 51).

### 5.2 Common method bias

This study employed Harman’s single-factor test to examine the presence of common method bias. According to this method, if the variance explained by the first unrotated factor is less than 40 percent, it suggests that common method bias is not a serious concern. An exploratory factor analysis was conducted on all measurement items, and nine factors were extracted. The first factor accounted for 24.926 percent of the total variance, which is below the 40 percent threshold. Therefore, it can be concluded that common method bias is not a significant issue in this study.

### 5.3 Measurement model assessment

#### 5.3.1 Reliability and convergent validity.

In this study, the Cronbach’s alpha coefficients for all latent variables exceeded 0.80, indicating strong internal consistency and high reliability. The factor loadings of all observed indicators on their respective constructs were above 0.70. In addition, the average variance extracted (AVE) values for all constructs were greater than 0.50, and the composite reliability (CR) values exceeded the recommended threshold of 0.70. These results collectively demonstrate that the measurement scales possess satisfactory convergent validity (Table 7).

**Table 7:** Cronbach’s α, AVE and CR values.

Latent Variable	Observed Variable	Factor Loading	Cronbach’s α	AVE	CR
CON	CON1	0.865	0.837	0.755	0.902
	CON2	0.863			
	CON3	0.878			
EFF	EFF1	0.892	0.86	0.782	0.915
	EFF2	0.873			
	EFF3	0.887			
EMO	EMO1	0.896	0.878	0.507	0.902
	EMO2	0.869			
	EMO3	0.878			
EPI	EPI1	0.879	0.856	0.776	0.912
	EPI2	0.873			
	EPI3	0.878			
POL	POL1	0.868	0.849	0.768	0.909
	POL2	0.87			
	POL3	0.864			
PS	PS1	0.851	0.85	0.572	0.889
	PS2	0.879			
	PS3	0.89			
PSY	PSY1	0.886	0.836	0.753	0.901
	PSY2	0.872			
	PSY3	0.868			
RI	RI1	0.858	0.845	0.763	0.906
	RI2	0.896			
	RI3	0.891			
SER	SER1	0.87	0.847	0.766	0.908
	SER2	0.884			
	SER3	0.888			
SOC	SOC1	0.884	0.878	0.506	0.902
	SOC2	0.871			
	SOC3	0.873			

#### 5.3.2 Discriminant Validity.

The results indicate that all Heterotrait–Monotrait Ratio (HTMT) values among the latent constructs are below the recommended threshold of 0.85, suggesting that the model demonstrates satisfactory discriminant validity (Table 8).

**Table 8:** HTMT values.

	CON	EFF	EMO	EPI	POL	PS	PSY	RI	SER	SOC
CON										
EFF	0.576									
EMO	0.163	0.205								
EPI	0.168	0.21	0.553							
POL	0.337	0.34	0.073	0.147						
PS	0.166	0.139	0.554	0.489	0.078					
PSY	0.378	0.459	0.124	0.055	0.587	0.098				
RI	0.225	0.191	0.485	0.42	0.071	0.484	0.108			
SER	0.118	0.172	0.56	0.576	0.184	0.526	0.136	0.435		
SOC	0.359	0.389	0.133	0.076	0.614	0.105	0.568	0.157	0.091	

### 5.4 Structural model evaluation

**Fig 4:**
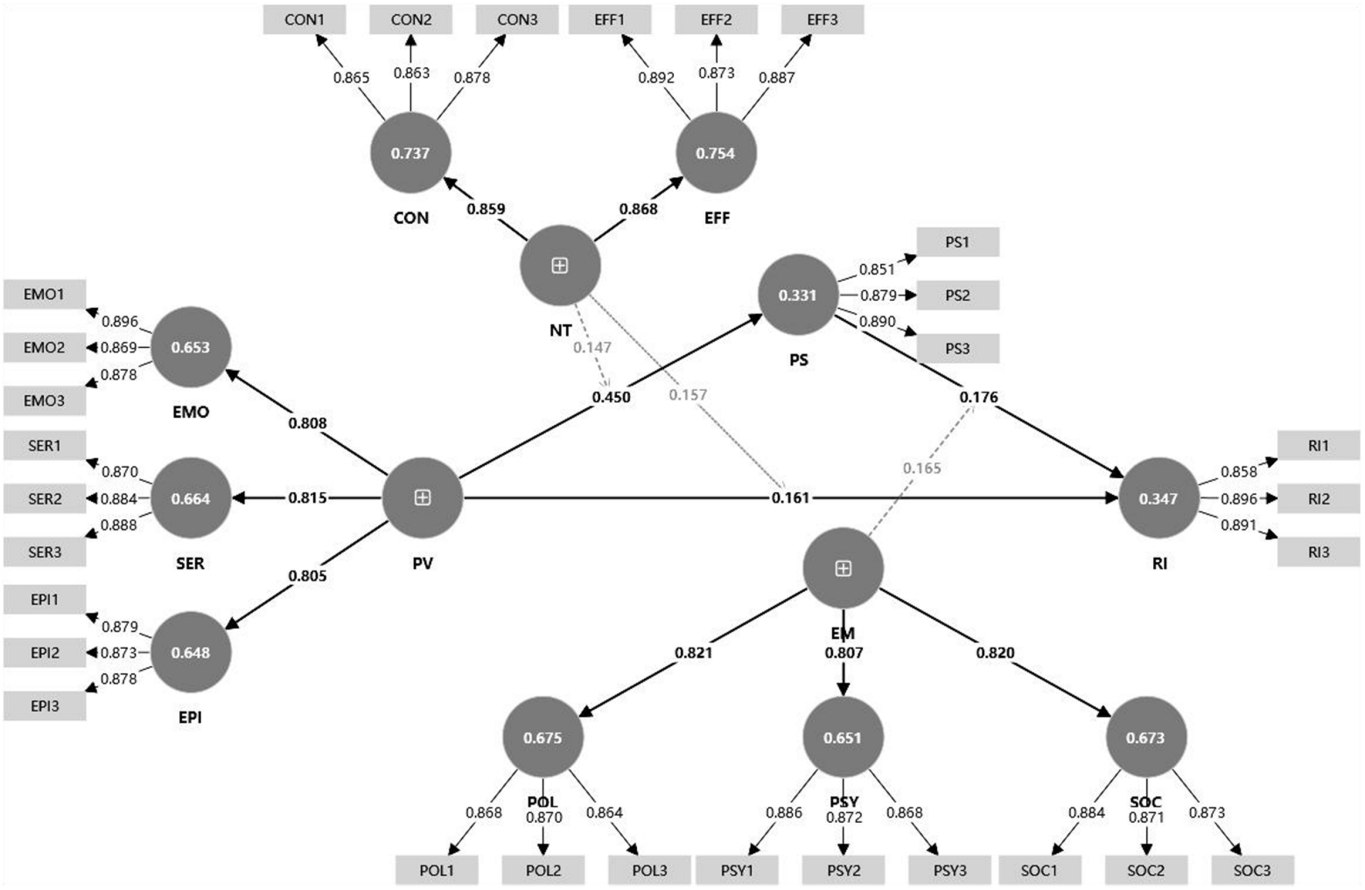
Structural equation modelinganalysis results.

This study employed SmartPLS 5 software to conduct structural equation modeling (SEM). The construction of the model and the results of its estimation are presented in Fig 4.

#### 5.4.1 Multicollinearity test.

This study assessed multicollinearity among the research variables using the Variance Inflation Factor (VIF). The results show that all VIF values were below the threshold of 5, indicating that multicollinearity is not a concern in this study (Table 9).

**Table 9:** Multicollinearity test.

	VIF
EM - > POL	1
EM - > PSY	1
EM - > RI	1.307
EM - > SOC	1
NT - > CON	1
NT - > EFF	1
NT - > PS	1.055
NT - > RI	1.319
PS - > RI	1.506
PV - > EMO	1
PV - > EPI	1
PV - > PS	1.492
PV - > RI	1.913
PV - > SER	1
NT x PV - > PS	1.428
NT x PV - > RI	2.116
EM x PS - > RI	1.945

#### 5.4.2 R² and F².

The results indicate that the R² values of all endogenous variables in the model exceed 0.26, suggesting that the exogenous variables have a good level of explanatory power for the endogenous constructs (Table 10). Furthermore, the F² values of the exogenous variables on the endogenous variables are all greater than 0.02, indicating that the exogenous constructs exhibit satisfactory explanatory effect sizes (Table 11).

**Table 10:** R².

	R Difference	Adjusted R²
CON	0.737	0.737
EFF	0.754	0.753
EMO	0.653	0.652
EPI	0.648	0.648
POL	0.675	0.674
PS	0.331	0.327
PSY	0.651	0.65
RI	0.347	0.34
SER	0.664	0.663
SOC	0.673	0.672

**Table 11:** f².

	f²
EM - > POL	2.074
EM - > PSY	1.864
EM - > SOC	2.055
NT - > CON	2.804
NT - > EFF	3.058
PS - > RI	0.031
PV - > EMO	1.882
PV - > EPI	1.845
PV - > PS	0.203
PV - > RI	0.021
PV - > SER	1.974
NT x PV - > PS	0.032
NT x PV - > RI	0.026
EM x PS - > RI	0.025

#### 5.4.3 Path analysis.

SmartPLS software was used to conduct path effect analysis in order to test the proposed research hypotheses. The results include standardized path coefficients (β), t-values, p-values, and 95% confidence intervals. Detailed results are presented in Table 12. The findings indicate that participant satisfaction (PS) has a significant positive effect on revisit intention (RI) (β = 0.185, p < 0.05). Perceived value (PV) also has a significant positive effect on participant satisfaction (PS) (β = 0.605, p < 0.05). In addition, perceived value (PV) has a significant positive effect on revisit intention (RI) (β = 0.186, p < 0.05). Therefore, Hypothesis H1 is supported.

**Table 12:** Path analysis.

	β	Se	t	p	5.00%	95.00%
PS - > RI	0.176	0.046	3.822	0	0.083	0.264
PV - > PS	0.45	0.047	9.632	0	0.355	0.537
PV - > RI	0.161	0.051	3.166	0.002	0.057	0.258

#### 5.4.4 Mediation analysis.

Mediation effects were tested using SmartPLS software. The results include the indirect effect value, standard error, t-value, p-value, and the 95% confidence interval. Detailed results are presented in Table 13.

**Table 13:** Mediation Analysis.

	β	Se	t	p	5.00%	95.00%
PV - > PS - > RI	0.079	0.023	3.456	0.001	0.035	0.126

The findings indicate that in the path “PV → PS → RI,” the mediating effect of participant satisfaction (PS) is 0.079. The 95% confidence interval does not contain zero, indicating that the mediation effect is statistically significant. Therefore, Hypothesis H2 is supported.

#### 5.4.5 Moderation analysis.

Moderation effects were tested using SmartPLS software. The results include interaction effect values, standard errors, t-values, p-values, and 95% confidence intervals. Detailed findings are presented in Table 14.

**Table 14:** Mediation effect analysis.

	β	Se	t	p	5.00%	95.00%
NT x PV - > PS	0.147	0.035	4.197	0	0.08	0.219
NT x PV - > RI	0.157	0.04	3.925	0	0.08	0.236
EM x PS - > RI	0.165	0.047	3.529	0	0.076	0.258

The interaction term between narrative transportation (NT) and perceived value (PV) has a significant effect on participant satisfaction (PS) (β = 0.147, p < 0.05), and the direct effect of PV on PS is positive (β = 0.450). This indicates that NT positively moderates the relationship between PV and PS, thus supporting Hypothesis H4.

The interaction term between NT and PV also shows a significant effect on revisit intention (RI) (β = 0.157, p < 0.05), with a positive direct effect of PV on RI (β = 0.161), indicating that NT positively moderates the relationship between PV and RI. Therefore, Hypothesis H3 is supported.

**Fig 5:**
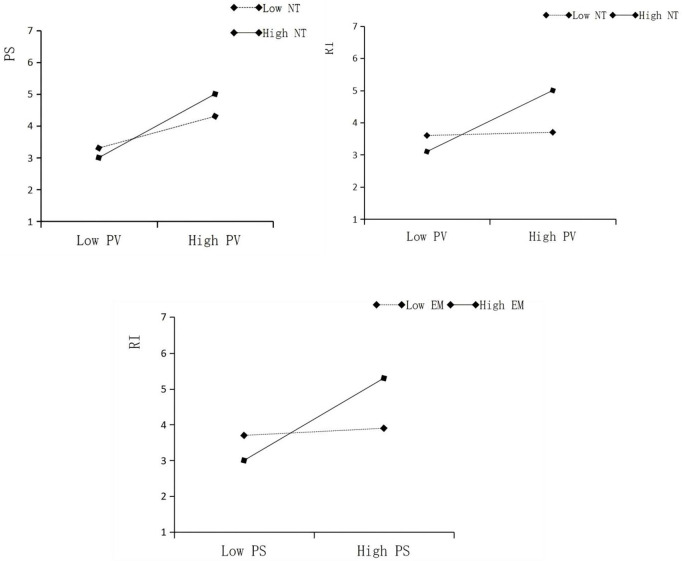
Moderation analysis.

Additionally, the interaction term between empowerment capability (EM) and participant satisfaction (PS) has a significant effect on revisit intention (RI) (β = 0.165, p < 0.05), and the direct effect of PS on RI is positive (β = 0.176). This suggests that EM positively moderates the relationship between PS and RI, thereby supporting Hypothesis H5 (Fig 5).

## 6 Discussion and conclusion

### 6.1 Discussion

Sustainable gardens—blending ecological aesthetics, environmental learning, and participatory design—operate as hybrid urban spaces where visitors co-create meanings through on-site interaction. Our results confirm a clear behavioral pathway: perceived value → satisfaction → revisit intention. In this context, perceived value is shaped less by utilitarian returns and more by intangible benefits—emotional pleasure, ideological resonance, and cognitive gains—thereby refining the classic value–satisfaction linkage in metropolitan, non-commercial settings [2,18].

We further show that narrative transportation functions as an in-situ psychological mechanism rather than a peripheral communication tool. When ecological and community stories are embedded in routing, interpretation, and participatory activities, immersion strengthens the translation of perceived value into both satisfaction and revisit intention. This moderating effect underscores the role of affective engagement and meaning-making in sustainability-oriented experiences and suggests that narrative design should enable identification and personalization [24–26].

In addition, empowerment capability—visitors’ perceived autonomy, voice, and influence—amplifies the satisfaction → revisit linkage. Visible co-creation roles and feedback-to-action loops help convert positive evaluations into loyalty, extending empowerment insights from resident-focused studies to visitor-side experience design within urban green settings [48].

Overall, the study integrates cognition (perceived value), affect (narrative immersion), and participation (empowerment) into a coherent account of how sustainable gardens cultivate loyalty. By aligning the research issue with these core concepts and demonstrating their empirical roles (mediated and moderated effects), the findings clarify the psychological–behavioral mechanisms through which sustainability meanings are internalized and sustained in contemporary urban tourism environments.

### 6.2 Theoretical implications

Grounded in the emerging context of urban sustainable gardens, this study contributes a behavior-oriented perspective to sustainable and community-based tourism by integrating cognitive evaluation (perceived value), affective immersion (narrative transportation), and participatory agency (empowerment capability) into a single explanatory framework for revisit intention. Prior research on sustainable initiatives has leaned toward infrastructure, spatial design, and environmental performance [5,6], while urban sustainable gardens have rapidly become laboratories for low-carbon, inclusive cultural practices [1,3,4]. By centering visitors’ cognitive–emotional–participatory experiences in these mission-driven, non-commercial spaces, our model advances a theoretical account of how sustainability meanings are perceived, internalized, and enacted through loyalty behavior in metropolitan settings.

First, we refine value-based explanations of tourist behavior by showing that, in sustainable gardens, perceived value is not primarily utilitarian but is strongly shaped by emotional and cognitive benefits—such as ideological resonance, learning gains, and aesthetic appreciation—thereby intensifying the classic value→satisfaction→revisit pathway [14,16]. This extends conventional findings from rural/commercial CBT contexts [7,49] to urban, hybrid sustainability spaces, where value is co-constructed through community interaction and place-based reflection [6,20].

Second, we reposition narrative transportation from a promotional device to an in-situ psychological mechanism that binds cognition and behavior. Immersion in environmental and community stories—delivered via interpretation and participatory activities—strengthens the translation of perceived value into satisfaction and revisit intention, clarifying how meaning-making operates within sustainability attractions [50]. The moderating role we identify specifies when perceived value most effectively converts into favorable outcomes: under high narrative immersion that catalyzes attentional focus, identification, and emotional resonance.

Third, by incorporating empowerment capability as a moderator of the satisfaction→revisit linkage, we extend empowerment theory from resident-centered assessments in CBT to visitor-centered experience design. Perceived autonomy, voice, and social influence in co-creative encounters amplify the behavioral payoff of satisfaction [2]. This conceptual move bridges empowerment and co-creation literatures, identifying empowerment as a psychological resource that consolidates loyalty in public, non-profit sustainability venues.

Taken together, the findings yield a perception–emotion–participation framework for sustainable tourism behavior in urban gardens: perceived value (cognition) fosters satisfaction, whose effect on revisit intention is jointly strengthened by narrative immersion (emotion) and empowerment (participation). The framework (1) addresses the documented gap between infrastructure-centric sustainability research and visitor behavior mechanisms, (2) delineates boundary conditions—urban, hybrid sustainability spaces featuring designed storytelling and participatory touchpoints (e.g., ecological display, community interaction, and experiential programs), (3) suggests testable extensions, including comparative studies across garden types and cities, additional mediators (e.g., identity, place attachment), and contextual moderators (e.g., prior environmental involvement) that can refine theory on how sustainability meanings are transformed into durable loyalty and pro-environmental intentions in metropolitan destinations.

### 6.3 Practical implications

Our findings offer actionable guidance for managers of sustainable gardens and urban CBT initiatives. First, because perceived value → satisfaction → revisit intention is the core pathway, managers should design experiences that elevate not only functional service quality but especially intangible value (emotional and cognitive gains). In practice, this means curating interpretive content that translates ecological processes into personally meaningful stories, and ensuring touchpoints (wayfinding, staff interactions, micro-learning stations) consistently deliver “aha” moments that visitors can articulate. In our survey, perceived value showed a significant positive effect on satisfaction and revisit intention; therefore, investing in interpretive quality and visitor care is likely to yield the highest loyalty returns.

Second, the significant moderating role of narrative transportation indicates that how stories are delivered on site matters as much as what they say. Gardens can operationalize this by: (a) weaving thematic narrative trails (e.g., “soil–seed–community” or “urban biodiversity recovery”) that connect exhibits and activities; (b) using guided storytelling and docents trained to prompt identification (“your role in this ecosystem”); and (c) deploying low-tech/tech-light media (QR-linked micro-stories, short audio clips, visitor-contributed anecdotes) to deepen immersion. In our data, higher narrative immersion strengthened the effects of perceived value on both satisfaction and revisit intention; thus, narrative design is a multiplier rather than a decorative add-on.

Third, because empowerment capability significantly strengthens the satisfaction → revisit link, managers should institutionalize participatory roles that give visitors real agency. Concrete steps include: (a) co-creation workshops (seed swapping, habitat making, exhibition prototyping) where visitor output is visibly incorporated on site; (b) micro-volunteering roles embedded in daily operations; (c) feedback-to-action loops (public boards or digital dashboards showing “your ideas implemented this month”); and (d) visitor councils or rotating “community curators” for seasonal programming. When visitors can see their input shaping the garden, satisfaction converts more reliably into repeat visits and advocacy.

Fourth, to broaden access and sustain engagement across metropolitan audiences, managers should build for inclusivity and continuity: offer multilingual micro-lessons, family-friendly and school-aligned modules, and return-visit incentives tied to learning progression (e.g., “Pollinator Passport” stamps, tiered badges). Because perceived value in our study included cognitive (learning) and emotional (alignment/joy) facets, programs that recognize learning progress and celebrate contributions are likely to raise both satisfaction and loyalty.

Finally, managers should track a compact set of implementation KPIs aligned with our model: (1) perceived value indices (emotional, cognitive, service), (2) narrative immersion scores for key programs, (3) empowerment markers (share of programs with co-created outputs; % visitor ideas implemented), and (4) behavioral outcomes (revisit intention, membership renewal, event re-enrollment). Running quarterly checks on these indicators allows teams to iterate narratives and participatory mechanisms where they have the largest marginal impact on satisfaction and revisits.

### 6.4 Limitations and future research

This study focuses on a single urban sustainable garden and uses non-probabilistic sampling, which limits statistical generalizability; future work should test the model across multiple cities/gardens with broader sampling. The cross-sectional, self-reported data constrain causal inference; longitudinal designs, behavioral indicators (e.g., actual revisits), and field experiments (e.g., narrative/empowerment interventions) are recommended. Our model centers on perceived value → satisfaction → revisit with two moderators; future studies could examine additional mechanisms (e.g., identity, place attachment) and contextual moderators (e.g., prior environmental involvement).

### 6.5 Conclusion

This study investigates revisit intention in sustainable gardens by integrating cognitive, emotional, and participatory perspectives. Using a mixed-methods design, we find that perceived value positively influences revisit intention through satisfaction. Narrative transportation strengthens the effects of perceived value on satisfaction and revisit intention, while empowerment capability amplifies the satisfaction–revisit link. These results clarify how environmental storytelling and participatory agency convert visitors’ value perceptions into loyalty in urban, mission-driven settings. Practically, gardens should prioritize interpretive quality, immersive narratives, and visible co-creation roles to increase return visits. Although based on a single site with non-probabilistic sampling, the proposed perception–emotion–participation framework offers a transferable basis for future multi-site, longitudinal, and experimental research on sustainable tourism experiences.

## Supporting information

S1 File Raw images. (PDF)
