# Supplementary material for: Exploring the mechanism by which tourists’ perceived value influences revisit intention in sustainable gardens: A case study of KICG-sustainable garden, Shanghai
Source: PLoS One. 2026 Jan 7;21(1):e0338508. doi: 10.1371/journal.pone.0338508 (PMC12779066; doi:10.1371/journal.pone.0338508)
Supplement: S1 File — (PDF) [file pone.0338508.s001.pdf]

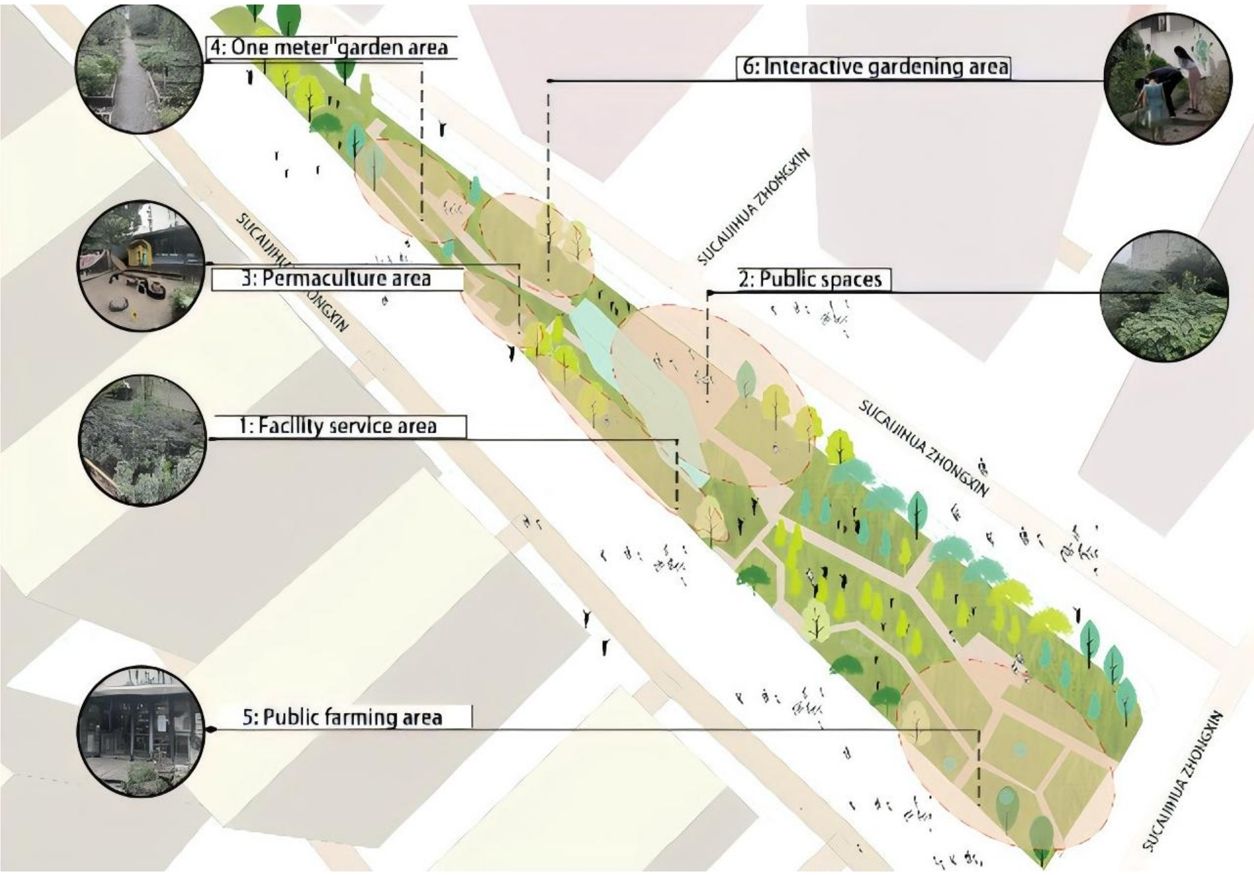

Figure1 KICG-Sustainable Garden (Created by the authors from field observation)

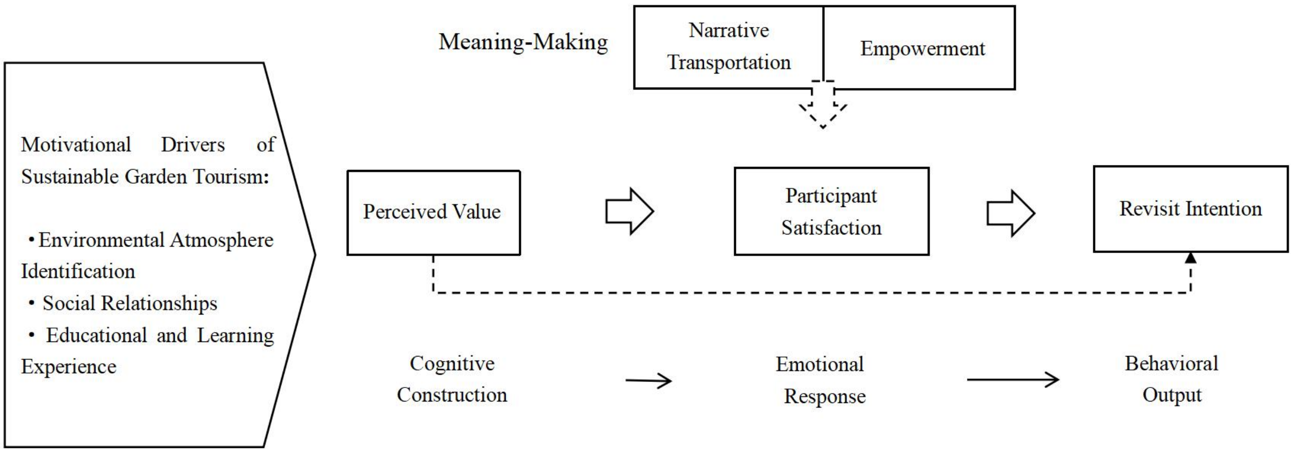

Figure 2. Theoretical Framework of the Influence of Tourists' Perceived Value on Revisit Intention in Sustainable Gardens

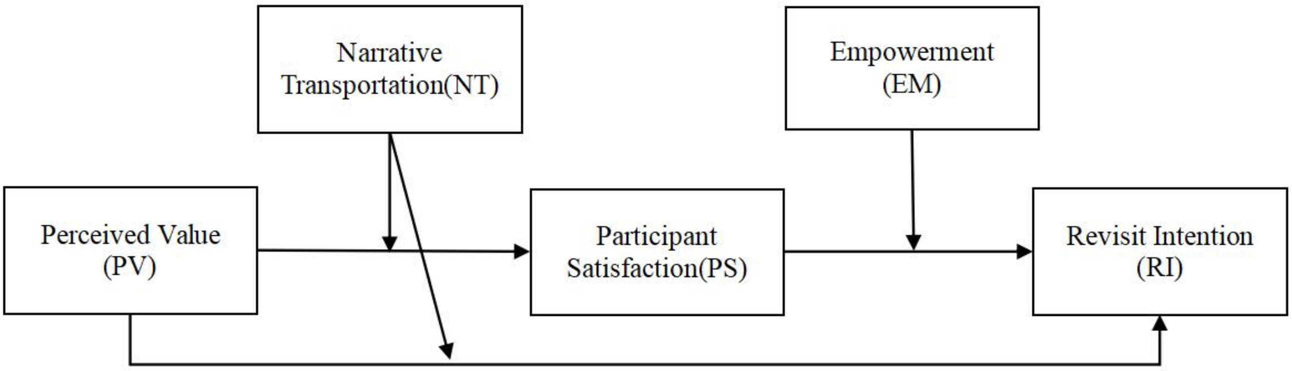

Figure3 model

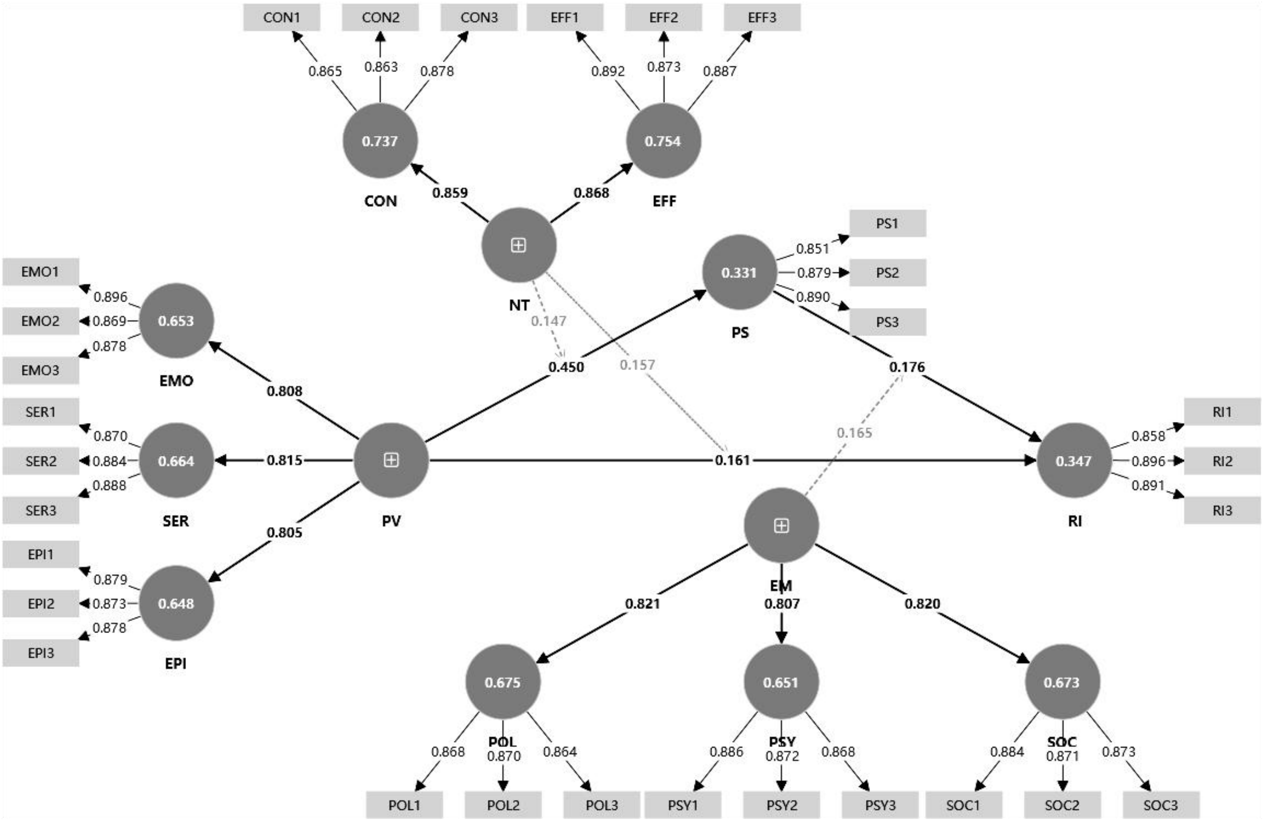

Figure4 Structural Equation Modeling Analysis Results

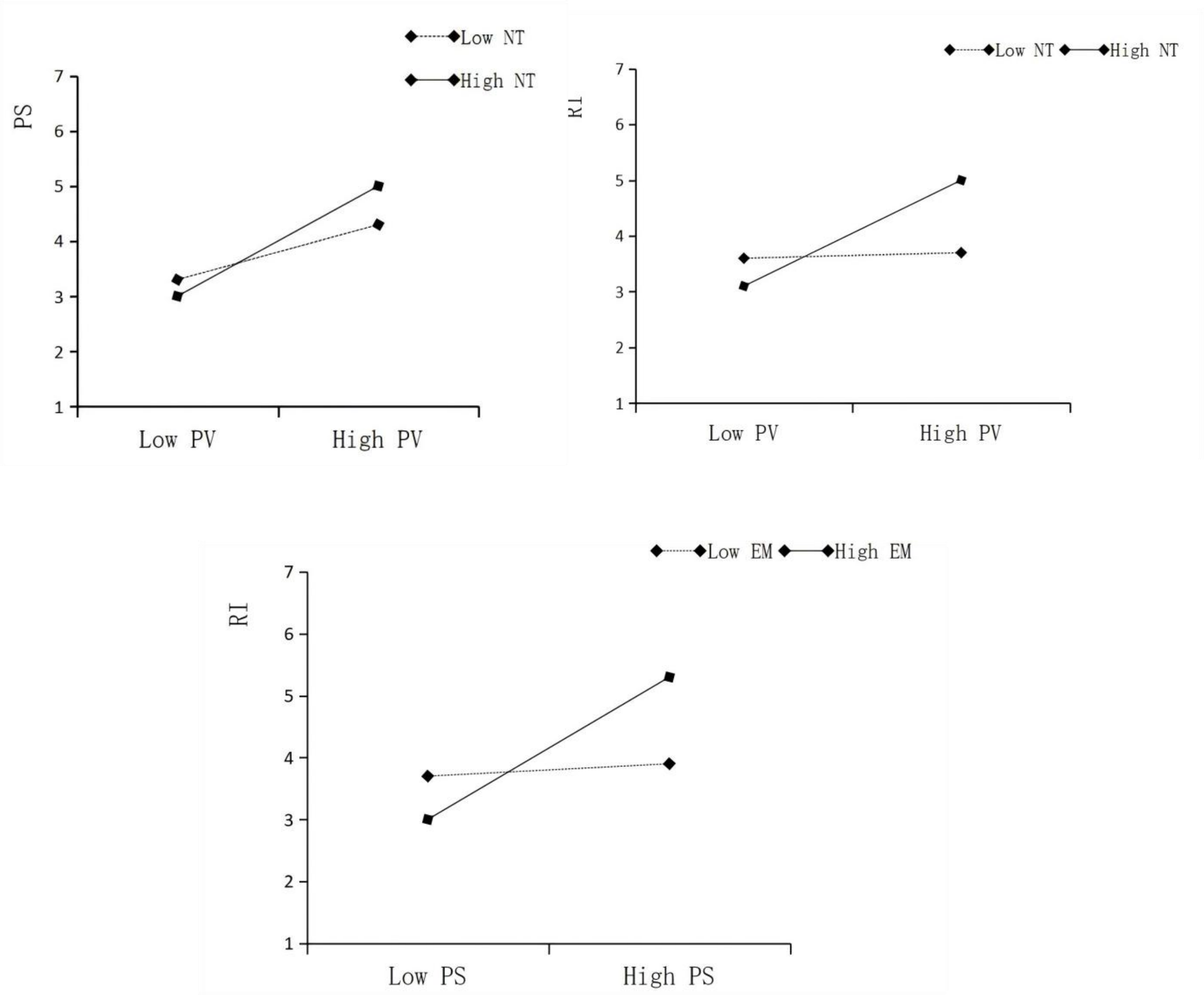

Figure5 Moderation Analysis
